# Contrast-enhanced CT-based radiomics model explained by the Shapley Additive exPlanations (SHAP) method for predicting preoperative diagnosis of pheochromocytoma and adrenal adenoma

**DOI:** 10.1186/s12880-026-02238-x

**Published:** 2026-02-26

**Authors:** Yiyao Li, Yao Yu, Peng Wu

**Affiliations:** Department of Urology, Nanfang Hospital, Southern Medical University, Guangzhou, Guangdong China

**Keywords:** Adrenal tumors, Radiomics, Machine learning, SHapley Additive exPlanations (SHAP)

## Abstract

**Objectives:**

This study aimed to develop and validate a prediction model that integrates radiomics with clinical characteristics, employing interpretable machine learning methods. The goal was to assist in the differential diagnosis of pheochromocytoma (PHEO) and adrenal adenoma, thereby providing a reference for decision-support information for patients with adrenal tumors.

**Methods:**

We retrospectively included 107 patients with PHEO and 230 patients with adrenal adenoma, all of whom were pathologically confirmed. Based on contrast-enhanced CT scans, we extracted 1,316 radiomics features and performed multiple rounds of feature selection to identify those with high discriminative relevance. Then we developed models incorporating these features along with clinical data, utilizing various algorithms including SVM, RF, SGD, KNN, XGBoost, and LightGBM. The diagnostic performance of these models was assessed using receiver operating characteristic (ROC) curves, Decision Curve Analysis, calibration curves, and DeLong tests. Finally, we used the SHapley Additive exPlanations (SHAP) method to interpret the contributions of different features.

**Results:**

The clinical-radiomics model demonstrated superior performance, achieving an area under the ROC curve (AUC) of 0.938. The Decision curve analysis (DCA) indicated that this model was more beneficial than either the clinical models or the radiomics models. Additionally, the SHAP method highlighted the contribution of each feature in the final model, with “log-sigma-1-mm-3D_glszm_GLNU” identified as the most important feature.

**Conclusions:**

Our study showed that the clinical-radiomics model using contrast-enhanced CT could effectively distinguish PHEO from adrenal adenomas.

**Supplementary Information:**

The online version contains supplementary material available at 10.1186/s12880-026-02238-x.

## Introduction

Adrenal tumors are rare, and they can be classified into several types, including adrenal adenomas, pheochromocytomas (PHEOs), and metastatic tumors. With advancements in imaging technology, especially computed tomography (CT), the detection rate of adrenal tumors has been increasing. Most of these tumors are asymptomatic and are often found incidentally during imaging for other reasons [1–4]. Histologically, PHEOs account for approximately 8% of adrenal tumors [5–7]. As tumors that secrete catecholamines, PHEOs can lead to symptoms such as hypertension, palpitations, dizziness, sweating, and even sudden death [8, 9]. Therefore, it is crucial to intervene promptly in patients diagnosed with pheochromocytoma. However, limited medical resources and a lack of clinical experience can result in misdiagnosis. Incorrect preoperative evaluation of pheochromocytomas can lead to serious complications during and after surgery, including hypertensive crises, refractory hypotension, and cortisol deficiency [10].

To confirm a diagnosis of PHEO, each patient must undergo a thorough clinical symptom assessment, imaging examinations, and endocrine hormone tests [7]. Hormone tests, including those measuring adrenocorticotropic hormone, cortisol, renin, aldosterone, epinephrine, and norepinephrine, require expensive equipment and skilled medical professionals. These tests can also be affected by food, medications, and timing, potentially leading to erroneous results [7, 9, 11]. Therefore, endocrine hormone tests may be more appropriate as a confirmatory measure for PHEOs.

Different adrenal tumors exhibit specific imaging features. For example, PHEOs typically show a CT plain scan density of 10 Hounsfield units (HU) or greater, while adenomas with high-fat content usually have a density of less than 10 HU. However, imaging diagnosis of PHEO is often complicated due to its varied appearances, which can include necrosis, fibrosis, cystic and fatty degeneration, and calcification. This variability is why PHEO is sometimes described as an “imaging chameleon” that can mimic other lesions [12–14]. CT scans are the preferred methods for evaluating adrenal lesions due to their speed, high resolution, and ability to enhance diagnostic accuracy when using contrast imaging. To assist in preoperative risk stratification of adrenal tumors, it is essential to enhance the diagnostic capabilities of CT in identifying PHEOs.

In recent years, radiomics has become well-known as a promising approach for extracting quantitative data from medical images. Radiomics can automatically and efficiently extract subtle patterns, textures, and biomarkers from medical images. Integrating radiomics with clinical practice holds promise for leading more informed decision-making and improving patient outcomes by providing non-invasive, quantitative information about tumor behavior, treatment response, or patient overall prognosis. Radiomics-based approaches are widely utilized in tumor staging, early diagnosis, differentiation, prognosis prediction, and treatment evaluation [15, 16]. Previous studies have demonstrated that machine learning algorithms leveraging radiomics features can enhance the discrimination between PHEO and adrenal adenoma, often yielding models with high predictive performance [5, 17]. Nevertheless, these studies lack interpretability regarding the internal mechanisms of the models, which may impede comprehension, diminish clinical trust in the decision-making process, and ultimately limit their reliability.

Various interpretation methods can improve the model’s interpretability, credibility, and reliability. Shapley Additive exPlanations (SHAP) is a widely used interpretation method that can clarify how each feature influences model outcomes, aiding clinicians in understanding the decision-making process [18, 19]. This study aims to construct and validate an interpretable radiomics model using multiple machine learning algorithms to differentiate PHEO from adrenal adenoma based on the radiomics features identified in contrast-enhanced CT scans. Our objective is to identify the simplest and most optimized model to improve the accuracy of preoperative diagnoses for adrenal adenoma and PHEO, and to enhance reliability through model interpretation.

## Methods

### Patient cohort

**Fig. 1:**
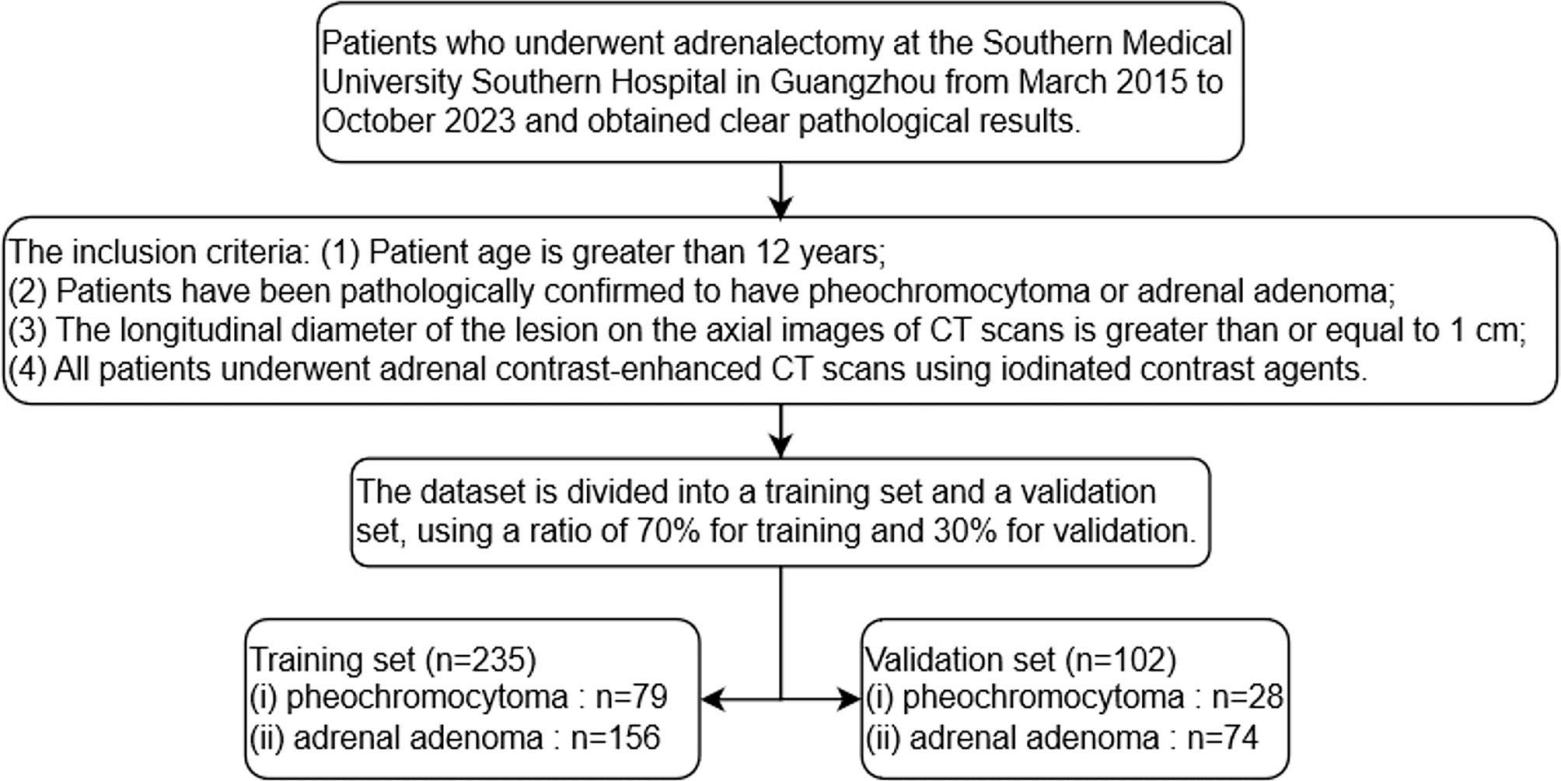
Patient recruitment process

The research subjects were sourced from Nanfang Hospital of Southern Medical University in Guangzhou. We identified consecutive patients who underwent adrenalectomy for adrenal tumors and obtained definitive pathological results between March 2015 and October 2023. The inclusion criteria for patients were as follows: (Ⅰ) age greater than 12 years; (Ⅱ) pathological confirmation of pheochromocytoma or adrenal adenoma; (Ⅲ) a longest diameter of the lesion, as observed on an axial CT scan, of at least 1 cm; (Ⅳ) patients must have undergone contrast-enhanced CT scans using iodinated contrast agents. Contrast-enhanced CT was selected as the primary imaging modality because it represents the standard imaging protocol for adrenal tumor evaluation in routine clinical practice. After the screening process, a total of 107 patients with PHEO and 230 patients with adrenal adenoma were selected. These individuals will be randomly divided into a training set of 235 cases and a validation set of 102 cases (Fig. 1). The research was approved by the Ethics Committee of Nanfang Hospital and was conducted in accordance with the Declaration of Helsinki (as revised in 2013). The requirement for informed consent was waived by the Ethics Committee because no identifying detail of the participants was included in this research.

### Sample size

Although formal sample size calculation is not well established for radiomics-based machine learning studies, we assessed the adequacy of our sample size using the Events per Variable (EPV) principle. In this study, 107 patients with PHEO (the smaller group) were included, and 11 variables (4 radiomics features and 7 clinical features) were ultimately selected for model construction, resulting in an EPV of approximately 9.7, which is considered acceptable for predictive modeling.

### Clinical characteristics

The clinical characteristics of the patients included in the study were as follows: age, gender, height, weight, body mass index (BMI), preoperative systolic and diastolic blood pressure, the longest diameter of the tumor measured via CT scan, as well as the presence of hypertension, diabetes, and smoking history. Additionally, symptoms such as headaches, palpitations, excessive sweating, and signs of Cushing’s syndrome (including moon face, central obesity, easy bruising, and thin extremities) were noted. Laboratory values included plasma cortisol levels at 8 a.m., supine plasma aldosterone levels, and serum potassium levels.

### CT protocol

Contrast-enhanced CT examinations were conducted at Nanfang Hospital using Siemens SOMATOM Definition AS CT scanners (Siemens, Germany). The scan parameters were configured as follows: tube voltage of 125 kV, automatic adjustment of mAs, a pitch of 0.9, X-ray tube rotation speed of 0.33 s per rotation, a field of view size of 320 mm × 320 mm, an acquisition matrix of 512 × 512, and a slice thickness of 0.625 mm. During image reconstruction, both the slice thickness and interval were set to 2 mm to ensure the acquisition of clear three-dimensional images. For the contrast-enhanced scan, a dual-chamber high-pressure injector was used to administer the contrast agent iohexol intravenously via the cubital vein at a concentration of 300 mg I/ml. The injection dose was controlled at 1.1 to 1.2 ml/kg of body weight, with an injection flow rate of 3.0 to 3.5 ml/s. All patients underwent non-contrast CT and three-phase contrast-enhanced CT scans, which were obtained at 20–25 s (arterial phase), 55–60 s (venous phase), and 180 s (delayed phase) after the administration of contrast material. The diagnosis of the adrenal mass was made by two radiologists (Radiologist A and Radiologist B). In cases of conflicting diagnostic opinions, a radiology professor with 20 years of experience in abdominal radiology was consulted to make the final determination.

### Tumor segmentation

**Fig. 2:**
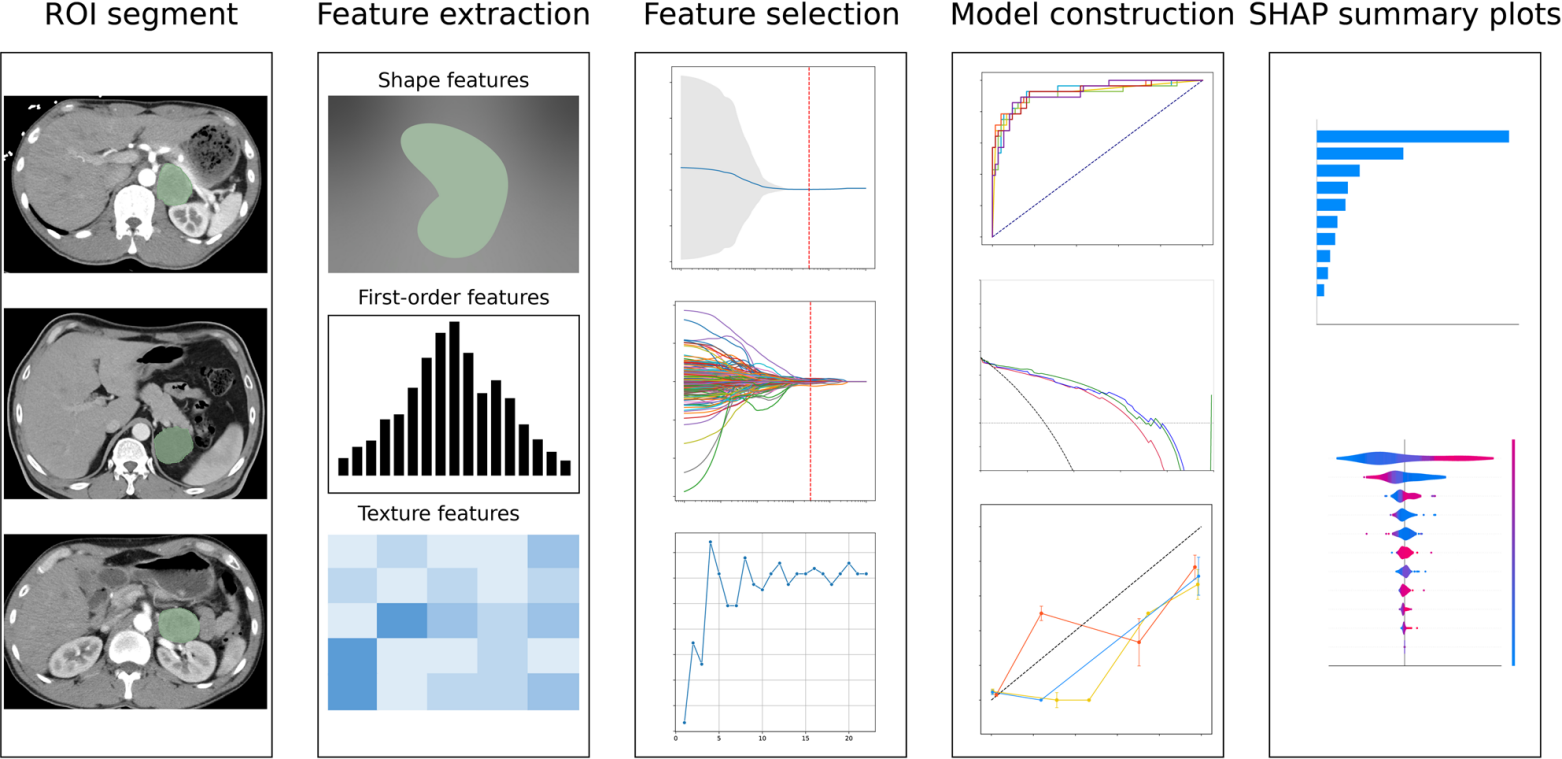
Technical workflow of this research

Images were obtained from the Picture Archiving and Communication System (PACS) and stored in the Digital Imaging and Communications in Medicine (DICOM) format. After removing any identifying information, such as patient names, manually delineate regions of interest (ROIs) for each patient’s contrast-enhanced CT images using the 3D Slicer software (version 5.2.2; Fedorov A. et al., 2012) [20]. Carefully identify each layer along the contour of the adrenal mass and manually trace the lesion margins while avoiding adjacent structures such as the kidneys, muscles, liver, and spleen. It is essential that the lesion area is fully delineated on each layer of the image (Fig. 2). To evaluate the consistency of lesion delineation, randomly select 20 cases and have two radiologists (A and B) independently delineate the lesions. Subsequently, extract radiomics features and calculate the intraclass correlation coefficients (ICC) to assess interobserver agreement. According to commonly accepted guidelines, an ICC value ≥ 0.75 indicates good to excellent reliability [21]. The evaluation criteria are as follows: ICC values ≥ 0.75 indicates no significant difference between the results, confirming passing consistency evaluation; ICC values of < 0.75 indicates inconsistent results.

### Radiomics feature extraction

Before performing radiomics feature extraction, the images must undergo preprocessing. The images are read using Python, and their grayscale values are normalized through Z-score standardization. This process helps reduce systematic errors that may arise from differences in CT scanner conditions across samples. In 3D Slicer, linear interpolation is applied to resample the images into isotropic voxels with a side length of 1 mm while preserving the slice thickness. The intensity values within the regions of interest (ROIs) are discretized at intervals of 25. For wavelet features, high-pass and low-pass filters are applied in the x, y, and z directions to each image, resulting in eight different decomposition combinations. In the case of Laplacian of Gaussian (LoG) features, 3D LoG filters are used to process the images, with σ values set to 5.0, 4.0, 3.0, 2.0, and 1.0 mm. This produces five derived images (Fig. 2). Finally, the Python PyRadiomics (version 3.1.0a2; van Griethuysen et al., 2017) package is utilized to perform radiomics feature extraction on the preprocessed contrast-enhanced CT images, yielding a total of 1,316 features [22].

### Feature selection and modeling

First, we need to perform feature selection to obtain a small set of features that exhibit strong independence and representativeness for model construction. We begin by using the Student t-test for preliminary screening to identify statistically significant features in the training set. Next, we conduct correlation analysis based on the K-Means clustering algorithm. For feature pairs with a similarity greater than 0.9, one feature from each pair is removed to enhance feature independence. Subsequently, we employ the least absolute shrinkage and selection operator (LASSO) regression to select the optimal predictive features from the training set. To prevent overfitting, we perform five-fold cross-validation on the training data. This involves dividing the dataset into five parts, using each part once as the validation set while the remaining four serve as the training set. We train and evaluate the model, repeating this process five times. The model that exhibits the best evaluation performance is selected as the final model. Features with non-zero weight coefficients obtained from the LASSO regression after lambda compression are retained. Finally, we conduct feature importance screening using the random forest algorithm. This algorithm determines the importance weights for each feature in the training set, which are then arranged in descending order of significance. Starting with the most important feature, we sequentially input each feature into the random forest algorithm to calculate grouped prediction probabilities and the area under the ROC curve (AUC). The AUC values corresponding to different numbers of features are statistically analyzed and plotted as a line graph. Based on the feature number-AUC line graph, we select the appropriate number of features and their corresponding attributes as the results (Fig. 2). To assess the robustness of feature selection, two additional independent methods (mutual information(MI) and recursive feature elimination(RFE)) were applied. Feature-level stability was evaluated using Jaccard index. Model-level stability was assessed by comparing the predictive performance on the validation sets.

The selected features are then imported into preset machine-learning algorithms to construct diagnostic models for PHEOs. These models include support vector machine (SVM), random forest (RF), stochastic gradient descent (SGD), K-nearest neighbors (KNN), eXtreme Gradient Boosting (XGBoost), and light gradient boosting machine (LightGBM). We use Bayesian hyperparameter optimization for the machine learning algorithms, with a randomly generated seed range of (200, 250). Model performance was assessed over 50 independent runs, and summary statistics are provided in the Supplementary Materials (Supplementary Table S1). For the hyperparameter optimization of each algorithm, we retain the training results that yield the highest ROC-AUC values.

### Statistical analysis

In our statistical analysis, we utilized several methods to evaluate clinical factors. Specifically, we employed independent samples t-tests, Mann-Whitney tests, and chi-square tests to identify clinical characteristics related to PHEO and adrenal adenoma. *P* < 0.05 was considered to be statistically significant. To assess the discriminatory power of our models, we used receiver operating characteristic (ROC) curves and the area under the ROC curve (AUC). We applied the DeLong test to compare ROC curves across different models. We quantified the discriminatory ability of our predictive models by calculating ROC-AUC, accuracy, F1 score, and Matthews correlation coefficient. Additionally, we performed decision curve analysis (DCA) to compute the net benefit (NB) of the models across various threshold ranges. Calibration curves were generated to evaluate the consistency between predicted probabilities and actual outcomes.

To interpret the feature importance and predicted probabilities from different machine learning algorithms, we applied the SHapley Additive exPlanations (SHAP) score. This involved importing features from the training set and computing SHAP values for each feature in the validation set. We visualized the correlation between feature importance and the relationship between features and predicted probabilities using violin plots for sample and feature SHAP values, as well as bar charts to display average SHAP values.

Data analysis and figure plotting were performed using the software Python (version 3.9.18), SPSS (version 25.0; IBM Corp., Armonk, NY, USA), and R (version 3.6.1).

## Results

### Patient characteristics

The clinical characteristics of the patients are summarized in Table 1. A total of 337 patients participated in the study, which included 107 diagnosed with PHEO and 230 with adrenal adenoma. The mean age of patients with PHEO was 47.69 ± 15.05 years, with 49.5% being male. In contrast, the mean age of patients with adrenal adenoma was 50.51 ± 10.83 years, with 47.4% being male. An intergroup comparison of clinical indicators between the PHEO and adrenal adenoma groups showed no statistically significant differences in gender, height, weight, BMI, hypertension, diabetes, smoking history, or symptoms of Cushing syndrome (*p* ≥ 0.05). However, there were statistically significant differences in age, preoperative systolic blood pressure, preoperative diastolic blood pressure, CT tumor longest diameter, headache, palpitations, sweating, plasma cortisol levels at 8 a.m., supine plasma aldosterone levels, and serum potassium levels (*p* < 0.05).

**Table 1:** The clinical baseline characteristics in patients with PHEO or adrenal adenoma

Characteristics	PHEO (*n* = 107)	Adrenal adenoma (*n* = 230)	*P* value
Gender, n (%)			0.40^(2)^
male	53(49.5)	109(47.4)	
female	54(50.5)	121(52.6)	
Age (years)	47.69 ± 15.05	50.51 ± 10.83	< 0.001^(1)﻿^
Height (cm)	163.04 ± 7.76	163.13 ± 7.89	0.773^(1)﻿^
Weight (kg)	58.20 ± 11.04	67.01 ± 13.22	0.138^(1)﻿^
BMI (kg/m²)	21.78 ± 3.17	25.05 ± 3.85	0.235^(1)﻿^
Preoperative SBP (mmHg)	132.29 ± 23 86	129 25 ± 15.41	< 0.001^(1)﻿^
Preoperative DBP (mmHg)	83.66 ± 16.22	81.42 ± 10.37	0.004^(1)﻿^
CT tumor LD (mm)	54.92 ± 27.78	20.34 ± 9.13	< 0.001^(1)﻿^
Hypertension, n (%)	72(67.3)	162(70.4)	0.322^(2)﻿^
Diabetes, n (%)	29(27.1)	45(19.6)	0.080^(2)﻿^
Smoking history, n (%)	25(23.4)	66(28.7)	0.186^(2)﻿^
Headache, n (%)	42(39.3)	54(23.5)	0.002^(2)﻿^
Palpitations, n (%)	39(36.4)	27(11.7)	< 0.001^(2)﻿^
Sweating, n (%)	18(16.8)	7(3.0)	< 0.001^(2)﻿^
Symptoms of Cushing syndrome, n (%)	0(0)	5(2.2)	0.146^(1)﻿^
Plasma cortisol at 8 a.m. (µg/dl)	16.11 ± 6.67	12.40 ± 4.72	0.012^(1)﻿^
Supine plasma ALD (ng/dl)	14.05 ± 8.77	22.51 ± 31.15	< 0.001^(1)﻿^
Serum potassium (mmol/L)	3.91 ± 0.38	3.62 ± 0.49	0.001^(1)﻿^

The data of age, height, weight, BMI, preoperative SBP, preoperative DBP, CT tumor longest diameter, plasma cortisol at 8 a.m., supine plasma ALD and serum potassium are presented as mean ± SD. ^(1)^ Independent sample T test; ^(2)^ Chi-square test. BMI, body mass index. SBP, systolic blood pressure. DBP, diastolic blood pressure. CT tumor LD, CT tumor longest diameter. ALD, aldosterone. SD, standard deviation

### Feature selection and model construction

**Fig. 3:**
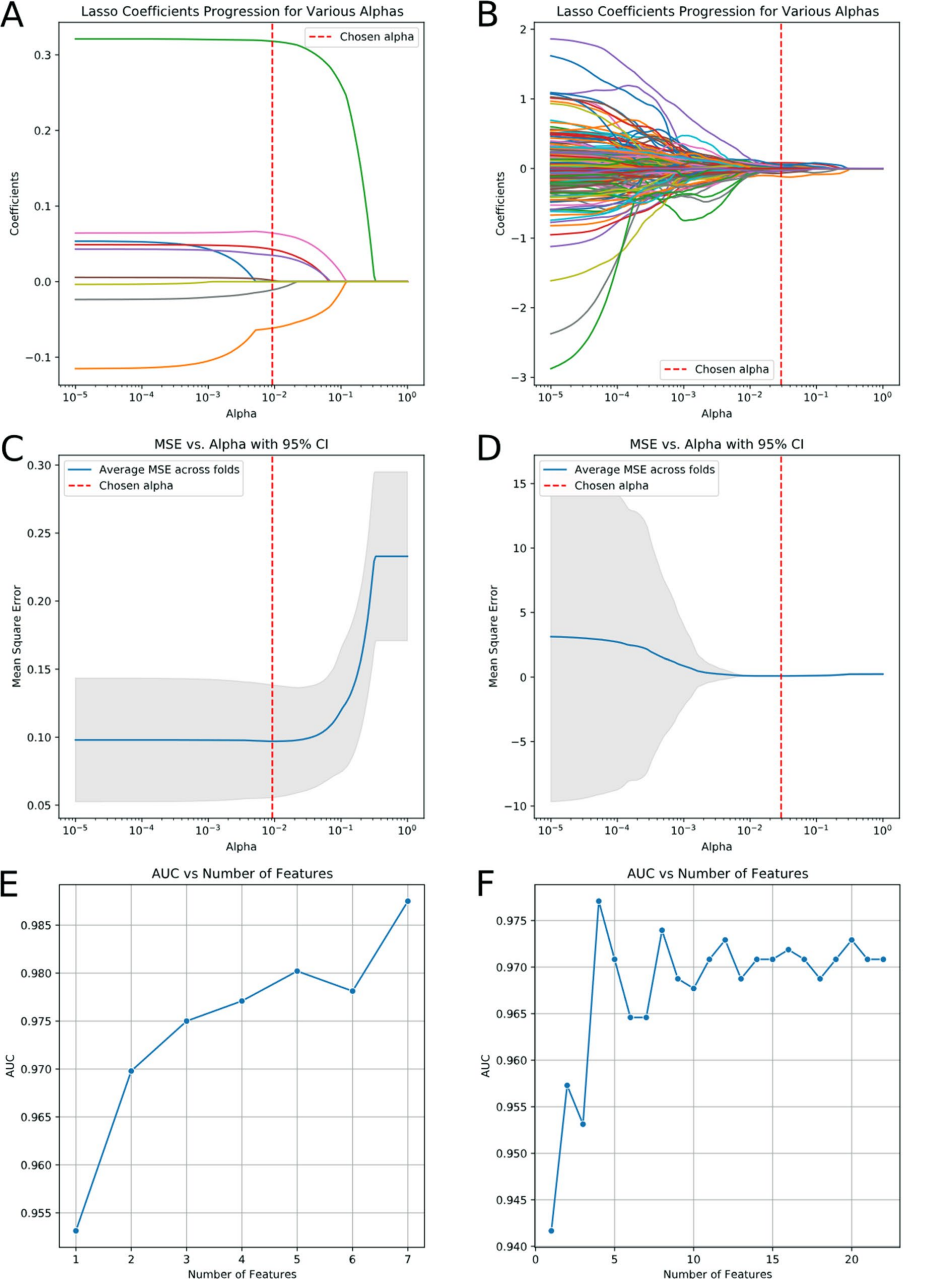
(**A-D**) Feature selection via the LASSO regression model. Features with nonzero coefficients were retained for the feature importance screening. (**D**,** E**) Line charts of Feature importance screening based on the relationship between the number of features and the AUC value. Figures A, C and E pertained to clinical features, whereas Figures B, D and F were focused on radiomics features

A total of 1,316 radiomics features were extracted from contrast-enhanced CT images. To reduce model complexity and enhance generalization ability, it was necessary to perform feature selection and dimensionality reduction. The feature selection process involved independent sample t-tests, correlation analysis using the K-Means clustering algorithm, LASSO regression, and feature importance screening based on the random forest algorithm (Fig. 3).

After completing the feature selection, 4 radiomics features were identified for model construction: “wavelet-LLL_glrlm_SRLGLE”, “log-sigma-1-mm-3D_glszm_GLNU”, “log-sigma-3-mm-3D_glszm_ZE” and “log-sigma-5-mm-3D_gldm_DV”. Additionally, 7 clinical features were selected for model construction: “BMI”, " CT tumor longest diameter”, “headache”, “palpitating”, “plasma cortisol at 8 a.m.“, " supine plasma ALD” and “serum potassium”. The Jaccard indexes between different feature selection methods ranged from 0.50 to 0.58, indicating moderate-to-good stability. The models achieved comparable predictive performance, with validation AUCs of 0.917, 0.932, and 0.922 for LASSO&RF-, MI-, and RFE-based models, respectively (Supplementary Table S2, S3).

Using both the radiomics and clinical features, as well as a combination of the two, we constructed predictive models employing SVM, RF, SGD, KNN, XGBoost, and LightGBM algorithms.

### Performance of prediction models

**Fig. 4:**
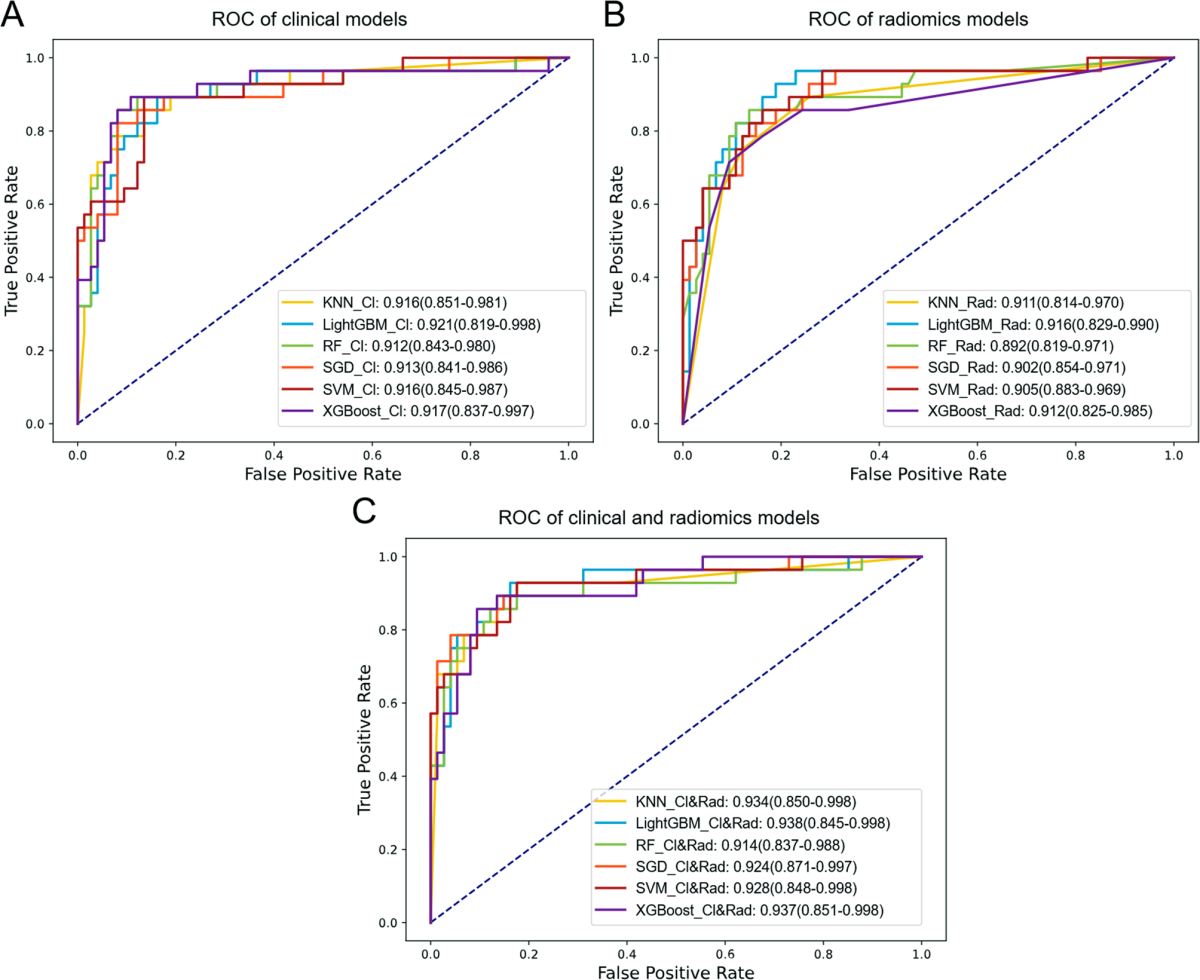
ROC curves comparison of predictive models, with AUC and 95%CI. (**A**) ROC curves of the clinical models. (**B**) ROC curves of the radiomics models. (**C)** ROC curves of the clinical and radiomics models

The ROC curves for the validation sets of different models are presented in Fig. 4, while the diagnostic performance data for these validation sets are shown in Table 2. For all six algorithms, an ablation study was performed to evaluate the contribution of different feature sets. Three models were constructed: clinical(Cl), radiomics(Rad), and combined models(Cl&Rad). The confusion matrix of the models in the validation set are presented in Supplementary Figure S1, S2 and S3, demonstrating balanced sensitivity and specificity.

The LightGBM models achieved the highest AUC value of 0.938 on the combined set (LightGBM_Cl&Rad) and the highest average AUC value of 0.925 on three feature sets. Furthermore, the LightGBM models performed well on other diagnostic metrics such as accuracy, F1 score, and Matthews correlation coefficient. Therefore, the LightGBM was being considered as the optimal algorithm.

**Table 2:** The comparison of diagnostic efficacy for the six prediction models on three validation sets

Model	Groups	AUC	Accuracy	F1-score	MCC	Average AUC
LightGBM	Cl	0.921	0.824	0.735	0.631	**0.925**
	Rad	0.916	0.853	0.754	0.655	
	Cl&Rad	**0.938**	**0.873**	**0.794**	**0.712**	
XGBoost	Cl	0.917	0.824	0.735	0.631	0.922
	Rad	0.912	0.853	0.717	0.62	
	Cl&Rad	0.937	**0.873**	**0.794**	**0.712**	
KNN	Cl	0.916	**0.873**	0.764	0.677	0.92
	Rad	0.911	0.853	0.737	0.635	
	Cl&Rad	0.934	0.853	0.746	0.644	
SVM	Cl	0.916	0.853	0.746	0.644	0.917
	Rad	0.905	**0.873**	0.787	0.702	
	Cl&Rad	0.928	0.853	0.746	0.644	
SGD	Cl	0.913	0.853	0.754	0.655	0.913
	Rad	0.902	0.863	0.774	0.684	
	Cl&Rad	0.924	0.863	0.767	0.673	
RF	Cl	0.912	0.824	0.735	0.631	0.906
	Rad	0.892	**0.873**	0.78	0.692	
	Cl&Rad	0.914	0.863	0.781	0.695	

Cl, clinical group. Rad, radiomics group. SVM, support vector machine.RF, random forest.SGD, stochastic gradient descent. KNN, K-nearest neighbors. XGBoost, eXtreme Gradient Boosting. LightGBM, light gradient boosting machine. AUC, area under the receiver operating characteristic curve. MCC, Matthews correlation coefficient

A comparative analysis using Delong tests on the ROC curves revealed that the only significant difference was between the LightGBM algorithm and the XGBoost algorithm; the remaining ROC curves did not show significant differences (Table 3).

**Table 3:** The comparison of prediction models by Delong tests

Models	LightGBM_Cl&Rad	LightGBM_Rad	LightGBM_Cl
SGD_Cl&Rad	0.658	0.388	0.296
SVM_Rad	1.000	0.495	0.535
LightGBM_Cl&Rad	1.000	0.454	0.519
SVM_Cl&Rad	0.875	0.632	0.490
XGBoost_Cl&Rad	0.770	0.878	0.522
KNN_Cl&Rad	0.730	0.912	0.531
LightGBM_Rad	0.454	1.000	0.763
XGBoost_Cl	0.744	0.980	0.293
KNN_Cl	0.691	0.915	0.719
RF_Cl	0.626	0.903	0.566
SVM_Cl	0.366	0.718	0.897
RF_Cl&Rad	0.216	0.655	0.918
SGD_Cl	0.362	0.691	0.906
SGD_Rad	0.139	0.382	1.000
LightGBM_Cl	0.519	0.763	1.000
RF_Rad	0.134	0.156	0.811
KNN_Rad	0.044*	0.087	0.291
XGBoost_Rad	0.027*	0.050	0.179

*, p value < 0.05, which is considered a statistically significant difference

**Fig. 5:**
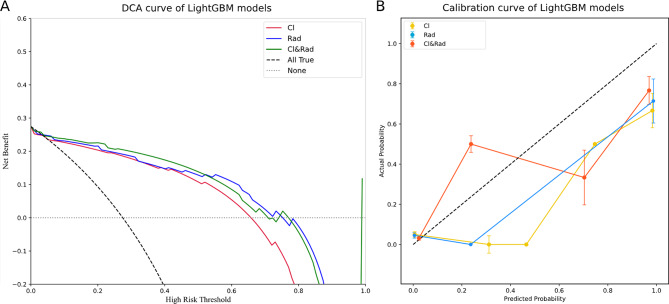
Comprehensive evaluation of the LightGBM models. (**A**) Decision curve analysis for the LightGBM models. (**B**) Calibration curve for the LightGBM models

The LightGBM model underwent further evaluation. As shown in Fig. 5, the DCA curve indicates that when the threshold probability is less than 0.52, the clinical-radiomics model provides greater benefits compared to both the clinical model and the radiomics model. Additionally, at threshold probabilities below 0.72, the clinical-radiomics model outperforms the “no treatment” strategy. The calibration curve further demonstrates that the clinical-radiomics model effectively identifies patients with PHEO and adrenal adenoma.

### Interpretability analysis based on SHAP

**Fig. 6:**
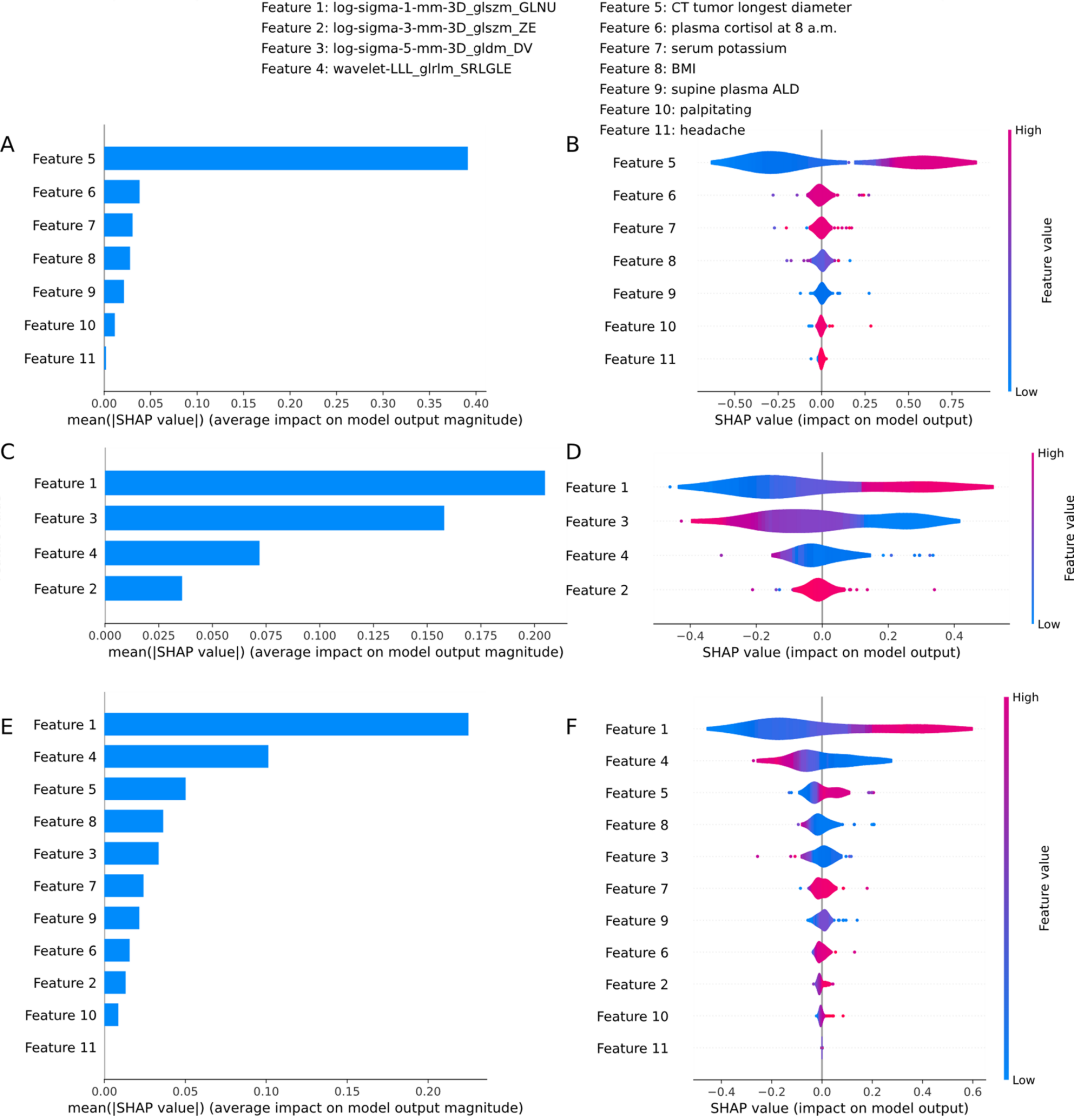
SHAP analysis of the LightGBM models. (**A**, **B**) Importance ranking plots of features in the clinical model. (**C**,** D**) Importance ranking plots of features in the radiomics model. (**E**,** F**) Importance ranking plots of features in the clinical and radiomics model

Using SHAP analysis, we examined the relationship between various features and predictive probabilities of PHEO and adrenal adenoma. We categorized these features as Features 1 through 11 (Fig. 6). Figures 6A, C, and E illustrate the ranking of feature importance, with Feature 1 identified as the most significant. In contrast, Feature 2, 6, 10, and 11 have a lesser impact on predictive probability. The feature correlation map reveals that in the clinical-radiomics model, Feature 3, 4, and 8 are negatively correlated with predictive probability, while Features 1, 5, 7, and 9 show positive correlation (Figs. 6B, D, and F). Additionally, for both clinical and radiomics models, Feature 5 is identified as the most important in the clinical model, whereas Feature 1 holds the highest significance in the radiomics model. This finding is consistent with the conclusions drawn from the clinical-radiomics model analysis.

## Discussion

In this study, we extracted radiomics features from contrast-enhanced CT image data and developed a predictive model that combines radiomics features with clinical risk factors using various machine learning algorithms to differentiate PHEO from adrenal adenoma [23].

PHEO is a rare neuroendocrine tumor that can cause symptoms such as hypertension, arrhythmia, or even cerebral hemorrhage due to excessive secretion of catecholamines [24]. While detecting elevated levels of catecholamines is a reliable method for diagnosing pheochromocytoma, its specificity is significantly influenced by preanalytical conditions. These conditions include the use of certain medications, such as dopamine D2 receptor antagonists, and the collection of 24-hour urine and blood samples [25, 26]. The cost of catecholamine testing is relatively high, and the variability in preanalytical conditions can lead to an increase in false-positive results. This may necessitate additional diagnostic tests to confirm or rule out PHEO at higher costs [27].

At present, biochemical testing and functional imaging constitute the standard diagnostic modalities for pheochromocytoma. Nevertheless, these procedures are associated with substantial costs, time-intensive, and not routinely administered to all patients presenting with adrenal incidentalomas. In contrast, the model developed in this study utilizes contrast-enhanced CT images, obviating the need for supplementary examinations or additional financial burden. The model facilitates rapid and non-invasive risk evaluation. Consequently, this model has the potential to serve as an adjunctive tool for preoperative risk stratification and for patient selection for subsequent endocrine assessment.

CT imaging is a common examination method used to diagnose adrenal tumors. In this field, several parameters are utilized to assess the benignity, malignancy, and functionality of adrenal lesions. These parameters include the maximum diameter of the tumor, HU value attenuation, the presence of regular margins, and both the relative and absolute clearance rates [28–34]. In recent years, advancements in radiomics technology have enabled the conversion of images into high-throughput quantitative features, providing more comprehensive information from the images [35–37]. Numerous studies have been conducted in the field of adrenal tumors focusing on applications such as differentiation, intraoperative risk assessment, and prognosis [34, 38–46]. For instance, Filippo et al. reported that radiomics features obtained from contrast-enhanced CT can distinguish between high-secretory and non-high-secretory PHEOs. Additionally, Jinhong Zhao et al. combined contrast-enhanced CT radiomics features with clinical features to develop predictive models that effectively stratify the preoperative risk of PHEOs [47].

This study integrates clinical features with radiomics features to develop a predictive model, which is then compared to clinical models and radiomics models. In the feature selection stage, we used LASSO regression and random forest algorithm. To evaluate the stability of feature selection, we used mutual information and recursive feature elimination as controls, and obtained good stability. Our stability analysis is consistent with previous studies that emphasize the use of multiple algorithms and statistical indicators to verify the importance of the feature selection process [48–53]. The observed consistency among different methods supports the robustness of the selected features. Although the three models did not show significant differences in their ROC curves, there are observable trends in their AUC values, accuracy, and F1 scores. Additionally, the DCA curve indicates that the clinical-radiomics model provides greater benefits. This finding underscores the potential value of the model for the preoperative diagnosis of PHEO.

Support vector machines and neural networks, along with other complex algorithms, are increasingly utilized to process high-throughput radiomics features to aid in diagnosis and predict treatment efficacy, showing superior performance. However, these models often lack transparency regarding how features contribute to prediction outcomes, a “black box” characteristic that impedes our understanding and trust in the decision-making processes of the models, thereby limiting their application in clinical practice [54, 55]. To address this challenge, SHAP as a method grounded in game theory, has been widely adopted in the field of radiomics for external explainability [56–58]. Through SHAP analysis, we identified that the most significant feature in clinical-radiomics models is log-sigma-1-mm-3D_glszm_GLNU, which is positively correlated with the prediction probability of PHEOs. GLSZM_GLNU refers to the grayscale non-uniformity of an area. For instance, high GLSZM_GLNU values typically indicate that a few highly significant components occupy a large volume within the tumor or tissue, such as extensive necrosis in PHEO. In contrast, low GLSZM_GLNU values are associated with uniform lipid components found in adenomas [59]. Therefore, for log-sigma-1-mm-3D_glszm_GLNU, a higher value may indicate extensive micronecrosis. The model explanations derived from SHAP analysis align well with the typical characteristics of PHEOs observed in CT images.

This study has several limitations that must be acknowledged. First, the majority of the included patients did not undergo MRI or PET-CT scans. Therefore, we only utilized contrast-enhanced CT for imaging examinations [60]. We acknowledged that multimodal imaging may further improve model performance. Second, due to the small number of patients with adrenal cortical carcinoma or adrenal metastatic tumors, these conditions were not represented in our study. Thirdly, differences in scanner vendors and acquisition parameters were not evaluated in this study, which may affect feature reproducibility. Then, this was a retrospective single-center study, which may limit the generalizability of our findings. Patient characteristics, imaging protocols, and scanner parameters may vary across institutions, potentially affecting model performance. Finally, the validation cohort was derived from the same institution, which may increase the risk of overfitting and optimistic performance estimation. Future studies will focus on prospective multicenter validation using external datasets from different institutions to further evaluate the robustness and clinical applicability of the proposed model.

## Conclusion

In summary, this study developed a predictive model using clinical symptoms and contrast-enhanced CT radiomics features to predict the presence of pheochromocytoma prior to surgical treatment, effectively distinguishing it from adrenal adenoma. This model can serve as a non-invasive tool to help clinicians determine whether patients require additional endocrine testing. However, further prospective validation of the model is necessary.

## Supplementary Information

Below is the link to the electronic supplementary material.

Supplementary Material 1

## Data Availability

The datasets used during the current study are available from the corresponding author on reasonable request.

## Abbreviations

CT: Computed tomography
SHAP: Shapley Additive exPlanations
PHEO: Pheochromocytoma
ROIs: Regions of interest
LASSO: Least absolute shrinkage and selection operator
MCC: Matthews correlation coefficient
ICC: Intraclass correlation coefficients
ROC: Receiver operating characteristic
AUC: Area under the receiver operating characteristic curve
MI: Mutual information
RFE: Recursive feature elimination
DCA: Decision curve analysis
EPV: Events per Variable
BMI: Body mass index
SBP: Systolic blood pressure
DBP: Diastolic blood pressure
ALD: Aldosterone
Cl: Clinical
Rad: Radiomics
SVM: Support vector machine
RF: Random forest
SGD: Stochastic gradient descent
KNN: K-nearest neighbors
XGBoost: eXtreme Gradient Boosting
LightGBM: Light gradient boosting machine
GLRLM: Gray-level run-length matrix
GLSZM: Gray-level size zone matrix
GLDM: Gray-level dependence matrix
