# Supplementary material for: Contrast-enhanced CT-based radiomics model explained by the Shapley Additive exPlanations (SHAP) method for predicting preoperative diagnosis of pheochromocytoma and adrenal adenoma
Source: BMC Med Imaging. 2026 Feb 26;26:183. doi: 10.1186/s12880-026-02238-x (PMC13067582; doi:10.1186/s12880-026-02238-x)

Supplementary Table S1

Mean, SD and CV of the Test AUC for 18 models in 50 optimizations

| Model | Mean | SD | CV |
| --- | --- | --- | --- |
| KNN_Cl | 0.907 | 0.010 | 1.10% |
| KNN_Rad | 0.886 | 0.008 | 0.91% |
| KNN_Cl&Rad | 0.918 | 0.004 | 0.46% |
| LightGBM_Cl | 0.896 | 0.027 | 3.03% |
| LightGBM_Rad | 0.905 | 0.007 | 0.80% |
| LightGBM_Cl&Rad | 0.928 | 0.008 | 0.82% |
| RF_Cl | 0.897 | 0.010 | 1.08% |
| RF_Rad | 0.888 | 0.004 | 0.43% |
| RF_Cl&Rad | 0.903 | 0.007 | 0.78% |
| SGD_Cl | 0.898 | 0.023 | 2.54% |
| SGD_Rad | 0.890 | 0.035 | 3.90% |
| SGD_Cl&Rad | 0.927 | 0.011 | 1.17% |
| SVM_Cl | 0.908 | 0.007 | 0.78% |
| SVM_Rad | 0.903 | 0.010 | 1.11% |
| SVM_Cl&Rad | 0.923 | 0.003 | 0.36% |
| XGBoost_Cl | 0.906 | 0.006 | 0.69% |
| XGBoost_Rad | 0.900 | 0.010 | 1.15% |
| XGBoost_Cl&Rad | 0.927 | 0.007 | 0.74% |

Supplementary Table S2

The Jaccard index between three different feature selection methods

|  | LASSO&RF vs MI | LASSO&RF vs RFE | MI vs RFE |
| --- | --- | --- | --- |
| Jaccard index | 0.500 | 0.579 | 0.583 |

Supplementary Table S3

The ROC-AUC values of models constructed using features selected by three different feature selection methods

| **Method** | **Train_AUC** | **Val_AUC** |
| --- | --- | --- |
| LASSO&RF | 0.984 | 0.917 |
| MI | 0.985 | 0.932 |
| RFE | 0.994 | 0.922 |

Supplementary Figure S1

The confusion matrices of the clinical models


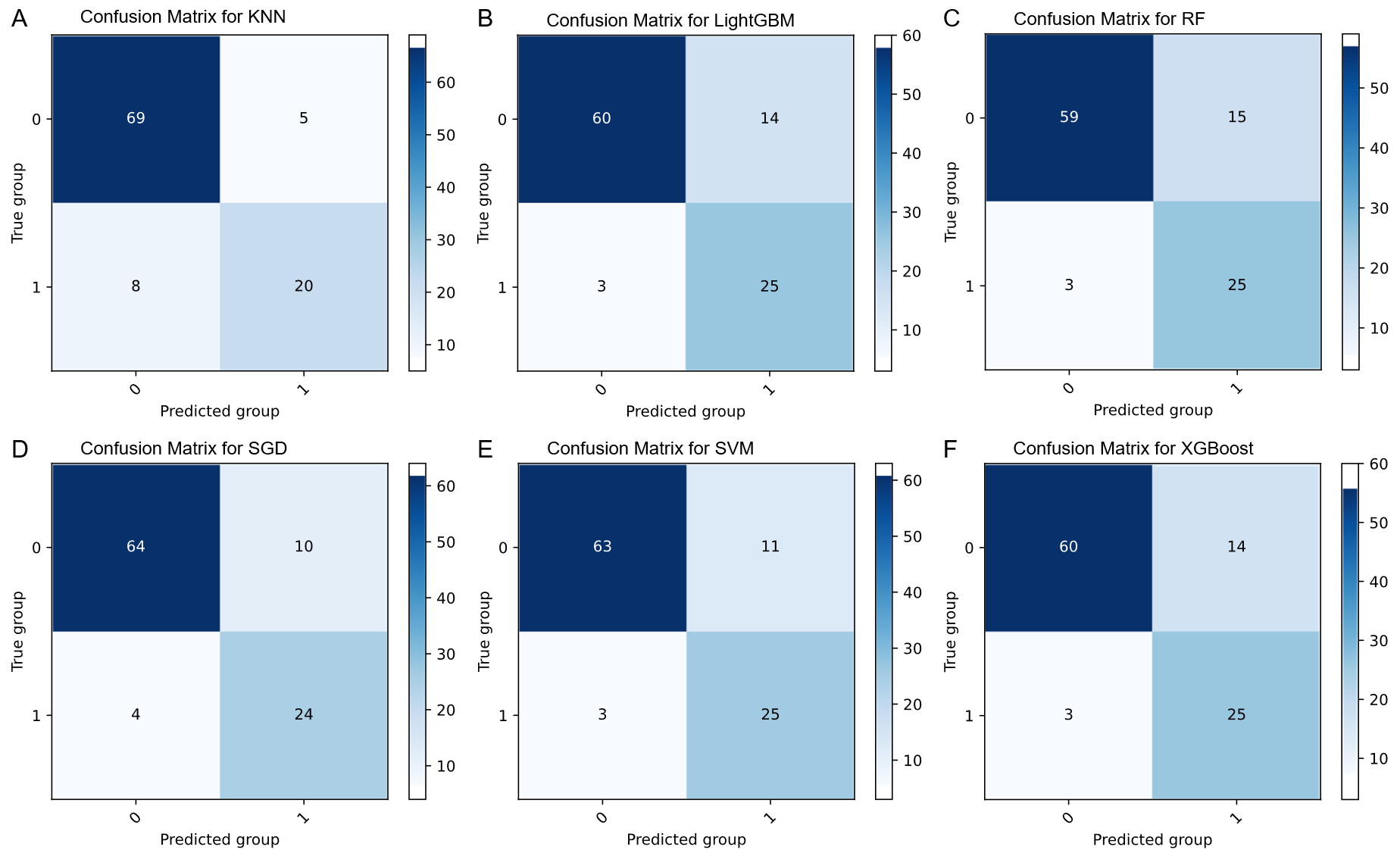


Supplementary Figure S2

The confusion matrices of the radiomics models


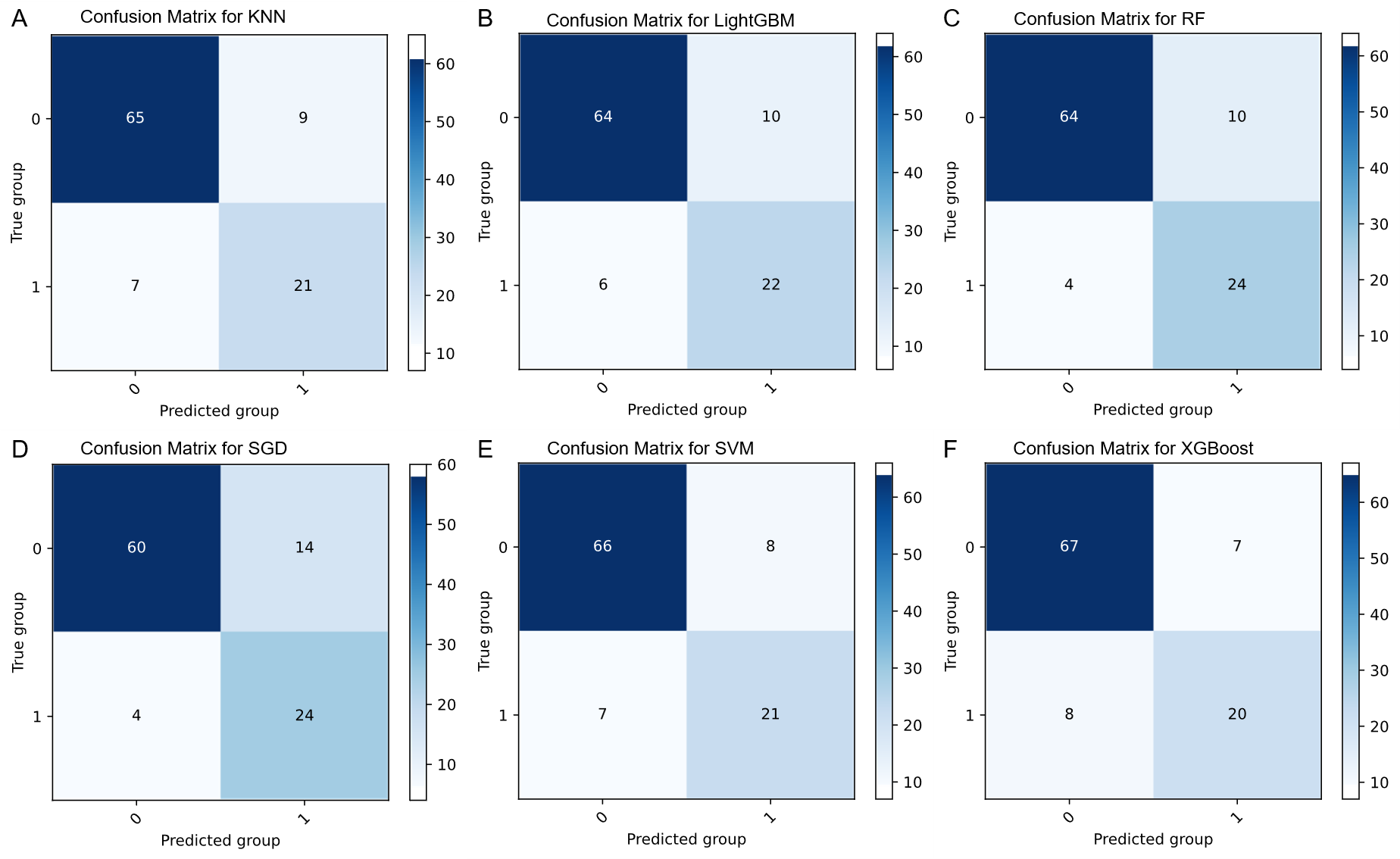


Supplementary Figure S3

The confusion matrices of the combined models


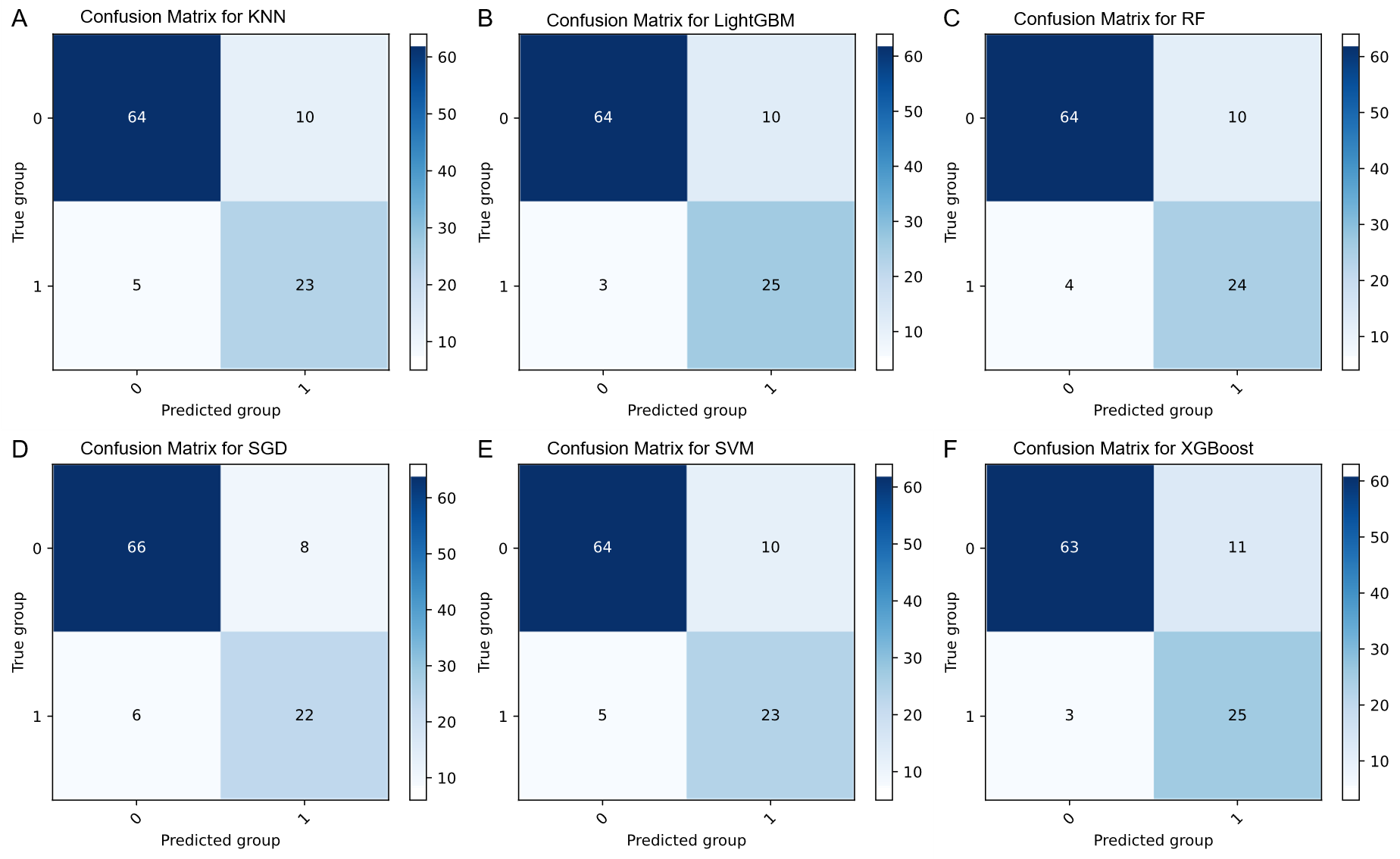

Supplement: Supplementary file 1 — Supplementary Material 1 [file 12880_2026_2238_MOESM1_ESM.docx]
